# A novel TRPV4 variant in spondylometaphyseal dysplasia, kozlowski type reveals a previously unreported loss-of-function mechanism

**DOI:** 10.1186/s13023-025-04070-y

**Published:** 2025-11-12

**Authors:** Han Wang, Shuang Li, Yiming Xu, Bin Feng, Xiuli Zhao, Xisheng Weng

**Affiliations:** 1 Department of Orthopedics, State Key Laboratory of Complex Severe and Rare Diseases, Peking Union Medical College Hospital, Chinese Academy of Medical Sciences & Peking Union Medical College, Beijing, 100730 China; 2 Center for Digital Medicine and Artificial Intelligence, National Infrastructures for Translational Medicine, Peking Union Medical College, Peking Union Medical College Hospital, Chinese Academy of Medical Science, Beijing, 100730 China; 3 Stem Cell Facility, Institute of Clinical Medicine, Peking Union Medical College Hospital, Chinese Academy of Medical Sciences, Beijing, 100730 China; 4 The Laboratory of Clinical Genetics, Medical Research Center, Peking Union Medical College Hospital, Chinese Academy of Medical Science & Peking Union Medical College, Beijing, 100730 China; 5 Rare Disease Medical Center, Peking Union Medical College, Peking Union Medical College Hospital, Chinese Academy of Medical Science, Beijing, 100730 China; 6 State Key Laboratory of Complex Severe and Rare Diseases, Institute of Basic Medical Sciences, School of Basic Medicine, McKusick-Zhang Center for Genetic Medicine, Chinese Academy of Medical Sciences, Peking Union Medical College, Beijing, 100005 China

**Keywords:** Spondylometaphyseal dysplasia, *TRPV4*, Bioinformatics analysis, Electrophysiology

## Abstract

Spondylometaphyseal Dysplasia, Kozlowski Type (SMDK) is an autosomal dominant skeletal disorder characterized by marked scoliosis, platyspondyly, overfaced pedicles, and mild metaphyseal changes. Pathogenic variants in TRPV4, which encodes a calcium-permeable nonselective cation channel, are known to underlie SMDK. In this study, we identified a previously unreported missense variant in NM_021625.5(*TRPV4*): c.2354G > C (p.Trp785Ser), in a patient clinically diagnosed with SMDK. This variant affects a highly conserved residue and is predicted to alter protein conformation. Functional validation through cellular experiments revealed that the p.W785S substitution markedly reduces agonist-induced calcium influx and membrane currents, indicating a loss-of-function effect on *TRPV4* channel activity. This deviates from the typical gain-of-function paradigm observed in most TRPV4-related skeletal dysplasias and may explain the relatively milder phenotype in our case. Our findings establish p.W785S as a novel pathogenic variant and highlight loss of TRPV4 activity as an alternative mechanism contributing to disease pathogenesis in SMDK.

**Supplementary Information:**

The online version contains supplementary material available at 10.1186/s13023-025-04070-y.

## Introduction

Intracellular calcium (Ca^2+^) signaling is a crucial second messenger system that regulates various cellular processes essential for homeostasis and responses to external stimuli. Changes in calcium concentrations are vital for numerous cellular functions across different tissue types, particularly in the musculoskeletal system [1]. The Transient Receptor Potential (TRP) superfamily of non-selective cation channels plays an integral role in Ca^2+^ regulation, with the transient receptor potential cation channel, subfamily V, member 4 (TRPV4) being a key mediator of calcium influx [2]. TRPV4 is polymodally modulated by various sensory stimuli, including temperature, osmotic pressure, and chemical agonists, which regulate its activity [3].

In mammals, TRPV4 is expressed predominantly in the musculoskeletal system, where it is involved in sensing mechanical forces and maintaining cartilage and bone integrity. Mutations in *TRPV4* lead to a spectrum of disorders, ranging from hereditary motor neuropathies to skeletal dysplasias [4–6]. Spondylometaphyseal Dysplasia, Kozlowski Type (SMDK), is an autosomal dominant disorder characterized by distinct phenotypic features, including shortened long bones, enlarged joints, pronounced kyphoscoliosis, and specific radiographic findings such as platyspondyly and metaphyseal widening [7]. TRPV4 mutations associated with SMDK are typically gain-of-function mutations, leading to abnormal calcium signaling in chondrocytes and defects in cartilage and bone development [8–10].

In this study, we identified a novel *TRPV4* mutation, c.2354G > C (p.Trp785Ser), linked to SMDK. This mutation occurs at a highly conserved site, and bioinformatic analysis suggested that it disrupted the protein’s secondary and tertiary structures, potentially altering channel function. To investigate how the mutation affected intracellular calcium dynamics, we overexpressed *TRPV4*-WT and *TRPV4*-W785S plasmids in HEK293 cells and compared their calcium signaling responses. Functional assays, including patch-clamp and Fluorometric Imaging Plate Reader (FLIPR) techniques, revealed that cells expressing the mutant *TRPV4* exhibited impaired calcium influx, with reduced responses to agonist stimulation compared to wild-type cells. These findings provided evidence for the pathogenicity of the p.W785S mutation and contributed to understanding how *TRPV4* mutations disrupt cellular signaling, ultimately leading to the development of SMDK.

## Materials and methods

### Subjects

The SMDK patient was recruited from the Peking Union Medical College Hospital (PUMCH) in 2021. This study was approved by the ethics committee of PUMCH and Institute of Basic Medical Sciences, Chinese Academy of Medical Sciences (IBMS, CAMS), and informed consent was obtained from the patient and his family. Clinical and genetic evaluations of the patient were performed by the clinicians and geneticists. The SMDK patient, a 23-year-old male from Shandong province in China, presented with his first symptom of spinal abnormalities at age 4–5 years. There was no hereditary history in his family of the disorder, and the parents of the proband were nonconsanguineous marriage, the patient had no affected siblings.

### Sequencing and bioinformatics analysis

Peripheral blood samples were collected from the proband and both parents for molecular genetic analysis. Whole-exome sequencing (WES) was performed on the proband using the Illumina platform (Illumina, USA) to identify potential disease-causing variants. Sequence reads were aligned to the human reference genome (GRCh37/hg19) and processed for variant calling using SAMtools (v1.9) to detect single nucleotide polymorphisms (SNPs) and small insertions/deletions (InDels). Variant annotation, including structural classification and functional prediction, was conducted using ANNOVAR (2017Jun08). Candidate variants were prioritized based on a minor allele frequency (MAF) of < 0.001 in population databases and their consistency with the proband’s clinical presentation. To confirm WES-detected variants and assess segregation within the family, polymerase chain reaction (PCR) amplification followed by Sanger sequencing was performed using an Applied Biosystems Genetic Analyzer (Applied Biosystems, USA). Sequence data were aligned to the *TRPV4* reference transcript (NM_021625.5) following the Human Genome Variation Society (HGVS) nomenclature guidelines. Genomic and coding reference sequences for *TRPV4* were obtained from the University of California, Santa Cruz (UCSC) Genome Browser. Pathogenicity predictions for candidate variants were performed using the VarCards platform, which integrates multiple in silico algorithms to assess potential deleterious effects.

### Homology modeling and structural analysis of mutant TRPV4 protein

The amino acid sequence of the mutant TRPV4 protein was obtained in FASTA format and submitted to the SWISS-MODEL web server (https://swissmodel.expasy.org) for homology modeling. Template identification was performed by searching the SWISS-MODEL template library (SMTL) to detect structural homologs based on sequence similarity and evolutionary relationships. Suitable templates were selected based on sequence identity, coverage, and experimental resolution of the template structures. Sequence-structure alignment was carried out to establish residue-level correspondence between the mutant TRPV4 sequence and the selected template. Homology model construction was performed using the SWISS-MODEL automated pipeline, which generates a three-dimensional structural model by satisfying spatial restraints and optimizing molecular geometry under energy minimization protocols. For regions lacking reliable alignment or absent in the template, loop modeling algorithms were applied to generate sterically and energetically plausible conformations. Model quality was evaluated using SWISS-MODEL’s integrated structure assessment tools, including Global Model Quality Estimate (GMQE), QMEAN scoring, stereochemical validation, and steric clash analysis. The resulting 3D model was downloaded for visualization and further structural analysis, including inspection of the mutation site, local conformational changes, and potential alterations in inter-residue interactions.

### Generation of *TRPV4*-WT and *TRPV4*-W785S constructs

The full-length human *TRPV4* coding sequence (NM_021625.5) was amplified and subcloned into the pmCherry-C1 expression vector. The missense variant (p.W785S) was introduced into the wild-type (WT) *TRPV4* construct by site-directed mutagenesis using mutation-specific primers and high-fidelity DNA polymerase (Takara, Shiga, Japan). The resulting *TRPV4*-WT and *TRPV4*-W785S plasmids were transformed into *E.coli* DH5α competent cells (Takara, Shiga, Japan) and cultured on LB agar plates supplemented with the appropriate antibiotic. Individual colonies were screened, and plasmid DNA was purified for sequence verification by Sanger sequencing to confirm the presence of the desired mutation and the absence of unintended sequence alterations. Verified clones were propagated in LB liquid medium under selective conditions, and large-scale plasmid preparations were obtained using the EndoFree Maxi Plasmid Kit (TIANGEN, China) according to the manufacturer’s instructions.

### Over expression of *TRPV4*-WT and *TRPV4*-W785S plasmids in HEK293 cells

Human embryonic kidney 293 (HEK293) cells were maintained in Dulbecco’s Modified Eagle Medium (DMEM; Gibco, USA) supplemented with 10% heat-inactivated fetal bovine serum (FBS; Gibco, USA) and 1% penicillin-streptomycin (Gibco, USA) at 37 °C in a humidified atmosphere containing 5% CO₂. For transient transfection, cells were seeded at a density of 5 × 10⁵ cells/well in 6-well plates approximately 24 h prior to transfection to achieve 70–80% confluence. Plasmid DNA (3 µg/well) encoding either *TRPV4*-WT or *TRPV4*-W785S was transfected using Lipofectamine 3000 reagent (Thermo Fisher Scientific, USA) following the manufacturer’s instructions.

### Quantitative real-time PCR and immunocytochemistry

Twenty-four hours after transfection, total RNA was extracted from HEK_*TRPV4*_ cells using TRIzol reagent (Invitrogen, USA) according to the manufacturer’s instructions. Complementary DNA (cDNA) was synthesized from 1 µg of total RNA using PrimeScript RT reagent kit (Takara, Japan). Quantitative real-time PCR (RT-qPCR) was performed using TB Green *Premix Ex Taq* Ⅱ (Takara, Japan) on CFX96 Real-Time PCR System (Bio-Rad, USA) to assess transfection efficiency and TRPV4 mRNA expression levels.

For immunocytochemistry, HEK293 cells were grown on sterilized glass coverslips and transfected with either *TRPV4*-WT or *TRPV4*-W785S constructs. At 48 h post-transfection, cells were washed three times with phosphate-buffered saline (PBS; without Ca²⁺ and Mg²⁺), fixed in 4% paraformaldehyde (PFA) for 15 min at room temperature, and permeabilized with 0.1% Triton X-100 for 15 min. Non-specific binding was blocked with 3% bovine serum albumin (BSA) for 1 h at room temperature. Cells were then incubated overnight at 4 °C with rabbit anti-TRPV4 primary antibody (Abcam, ab191580) diluted to 5 µg/mL in blocking buffer. Following three PBS washes, cells were incubated with Alexa Fluor™ 594-conjugated donkey anti-rabbit IgG secondary antibody (Invitrogen, A32740) for 1 h at room temperature in the dark. After three final PBS washes, nuclei were counterstained with 4′,6-diamidino-2-phenylindole (DAPI; Invitrogen, USA) for 5 min. Coverslips were mounted using antifade mounting medium, and images were acquired with a Leica TCS SP8 STED confocal microscope (Leica Microsystems, Germany) to evaluate TRPV4 protein expression and subcellular localization.

### Intracellular Ca^2+^ measurements

Intracellular Ca²⁺ flux was quantified using a Fluorometric Imaging Plate Reader (FLIPR) assay with the Calcium 6 Assay Kit (Bulk Kit; Molecular Devices, R8191) following the manufacturer’s protocol. Twenty-four hours post-transfection, HEK_*TRPV4*_ cells were detached using 0.25% trypsin-EDTA (Gibco, USA) and seeded into black-walled, clear-bottom 384-well plates (Corning, USA) at a density of 1 × 10⁴ cells/well. On the day of the FLIPR assay (48 h post-transfection), the culture medium was replaced with loading buffer consisting of Hank’s Balanced Salt Solution (HBSS; Gibco, 14025-092) supplemented with 20 mM HEPES (ChemCruz, sc-29097 A) and the proprietary Calcium 6 dye. Plates were incubated at 37 °C in the dark for 2 h for dye loading. Agonist solutions were prepared in assay buffer containing 0.1% dimethyl sulfoxide (DMSO) as a vehicle control. The TRPV4 agonist GSK1016790A was serially diluted (three-fold) from a 10 µM starting concentration to generate 11 dose points, each tested in duplicate, and transferred into a compound source plate. Calcium flux was measured using the FLIPR Penta System (Molecular Devices, USA), fluorescence was measured at 515–575 nm, and data were expressed as the ratio of peak fluorescence intensity over baseline, and concentration-response curves were generated. Half-maximal effective concentration (EC₅₀) values were determined by non-linear regression curve fitting using GraphPad Prism version 8.0 (GraphPad Software, USA).

### Electrophysiology

Whole-cell patch-clamp recordings were performed to assess TRPV4-mediated membrane currents in HEK_*TRPV4*_ cells. Twenty-four hours post-transfection, cells were detached with 0.25% trypsin-EDTA (Gibco, USA) and seeded onto sterile glass coverslips (8 × 10³ cells/well) placed in 24-well plates. Coverslips were transferred to a continuously perfused recording chamber mounted on the stage of an inverted microscope (Mshot/Olympus, Japan). Patch pipettes were fabricated from borosilicate glass capillaries (Sutter Instruments, USA) using a micropipette puller (Sutter Instruments, USA), with resistances of 2–5 MΩ when filled with internal solution. The pipettes were positioned using a micro-manipulator (Scientifica/Sutter Instruments, USA) to gently contact the cell membrane. Negative pressure was applied to form a high-resistance (≥ 1 GΩ) seal. Following rapid capacitance compensation, additional suction was applied to rupture the membrane, establishing whole-cell configuration. Slow capacitance and series resistance were subsequently compensated; no leak subtraction was applied. Membrane potential was held at -80 mV. Current-voltage (I-V) relationships were obtained by stepping from 0 mV to -100 mV for 1 ms, followed by a 200 ms ramp to + 100 mV, repeated every 5 s. Currents were recorded using an EPC-10 amplifier (HEKA Elektronik, Germany) and PatchMaster software (HEKA Elektronik, Germany). The extracellular solution contained (in mM): 140 NaCl, 3.5 KCl, 1 MgCl₂·6 H₂O, 2 CaCl₂·2 H₂O, 10 D-glucose, 10 HEPES, 1.25 NaH₂PO₄·2 H₂O; pH adjusted to 7.4 with NaOH. The pipette (intracellular) solution contained (in mM): 50 CsCl, 10 NaCl, 10 HEPES, 60 CsF, 20 EGTA; pH adjusted to 7.2 with CsOH. All recordings were performed at room temperature. In HEK_*TRPV4*−WT_ and HEK_*TRPV4*−W785S_ cells, extracellular fluids containing 10 nM agonist GSK1016790A, 10 nM agonist GSK1016790A with 300 nM blocker GSK2193874, and fluids without compounds were respectively subjected to gravity perfusion through a recording bath to act on the cells, and liquid exchange was carried out using a vacuum pump. For each condition, recordings were obtained from three independent cells.

## Results

### Clinical manifestations

**Fig. 1:**
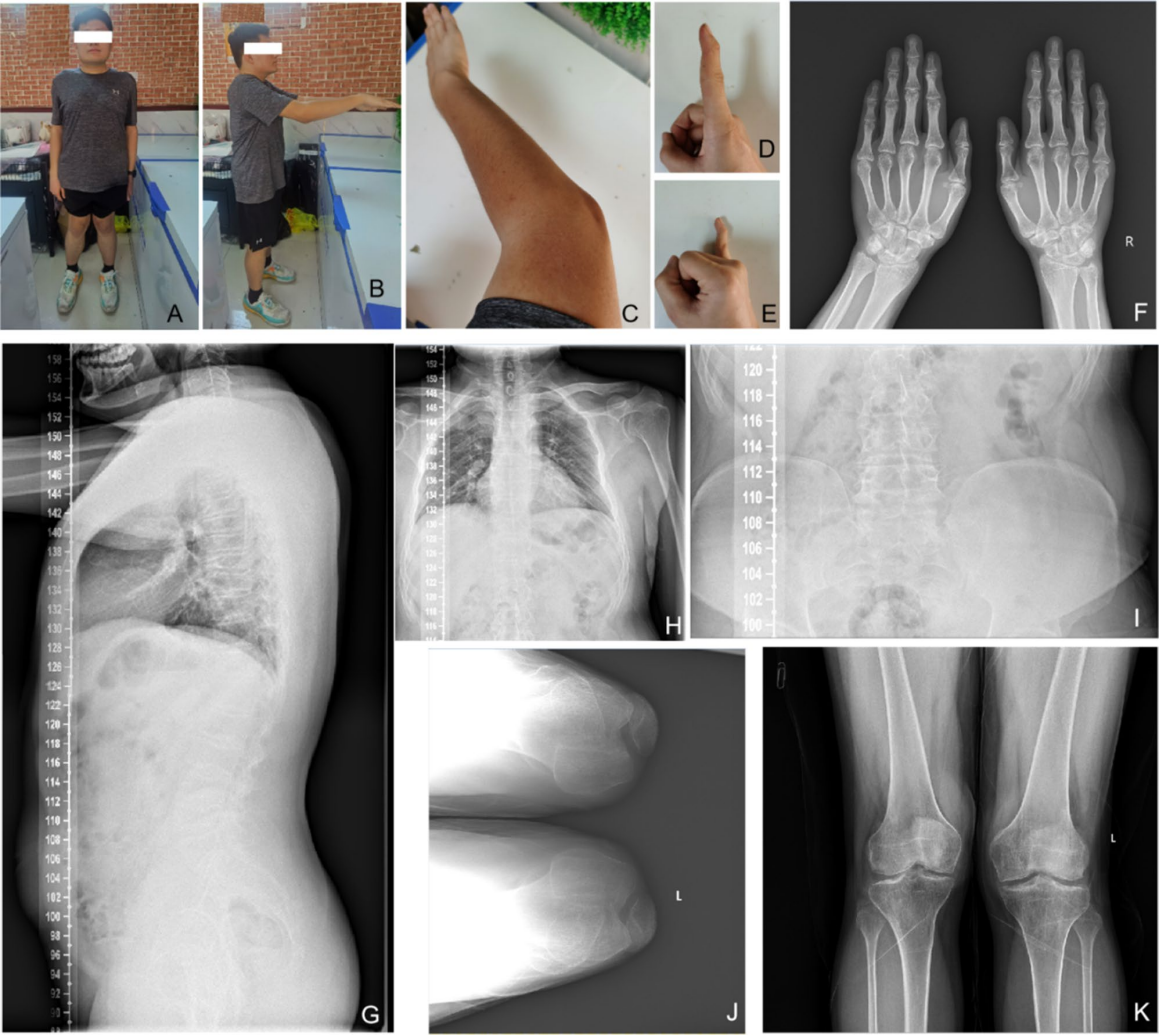
Clinical and radiological phenotypes of the patient with SMDK. **A**-**B**. The gross appearance of patient. **C**-**E**. The gross appearance of right upper limb (**C**), index finger (**D**) and little finger (**E**), showing the flexion deformity of joints. **F**. The AP radiograph of hands, showing the degeneration of small joints. **G**-**I**. The AP and LAT radiograph of spine, showing slight scoliosis, narrowed vertebral bodies, pectus carinatum, and inwardly curved scapulae. **J**. The axial radiograph of patellar. **K**. The AP radiograph of knees, showing degeneration of joints and genu varum of left knee

The proband is a 23-year-old male, 175 cm tall, and weighing 86 kg. The proband’s father has a height of 177 cm, and the mother has a height of 157 cm. The proband started walking later than peers and exhibited a waddling gait while walking. From the age of four or five, the proband developed a habit of hunching and bending the waist, and showed a lack of interest in physical activities. At around 12 years old, the proband experienced knee joint deformity and sought medical attention due to significant pain. By the age of 14, the pain worsened due to weight gain. Currently, after losing weight, the pain has reduced, and the proband can engage in normal activities. However, prolonged sitting or standing is uncomfortable, and conscious efforts have been made to correct the walking posture, resulting in less noticeable waddling gait. The main phenotypes observed are knee joint deformity, along with pain in the knee joints, spine, and elbow joints. Hip joint pain is not prominent, but standing after prolonged sitting causes discomfort. The wrist joints are not painful but may produce cracking sounds. The first joints of both index and little fingers are bent and cannot be straightened. Shoulder pain is not prominent but discomfort is felt when lying flat at night. In the past year, the proband has experienced pain when holding the head with hands and lifting objects. The neck exhibits lateral movement with pain, and there is occasional numbness in the fingers, possibly due to nerve compression caused by narrowed vertebral bodies. The respiratory function appears normal under normal circumstances, but after intense physical activity, insufficient lung capacity due to chest deformity leads to pain. There is no muscle atrophy observed. Rheumatoid-related blood indicators are within normal ranges, ruling out ankylosing spondylitis. X-ray results show slight scoliosis, narrowed vertebral bodies, pectus carinatum, and inwardly curved scapulae, which do not significantly affect daily life. The proband has an X-shaped leg posture when standing, restricted elbow joint movement, and limited mobility (Fig. 1).

### Validation and functional analysis of a *TRPV4* missense mutation detected through whole-exome sequencing

**Fig. 2:**
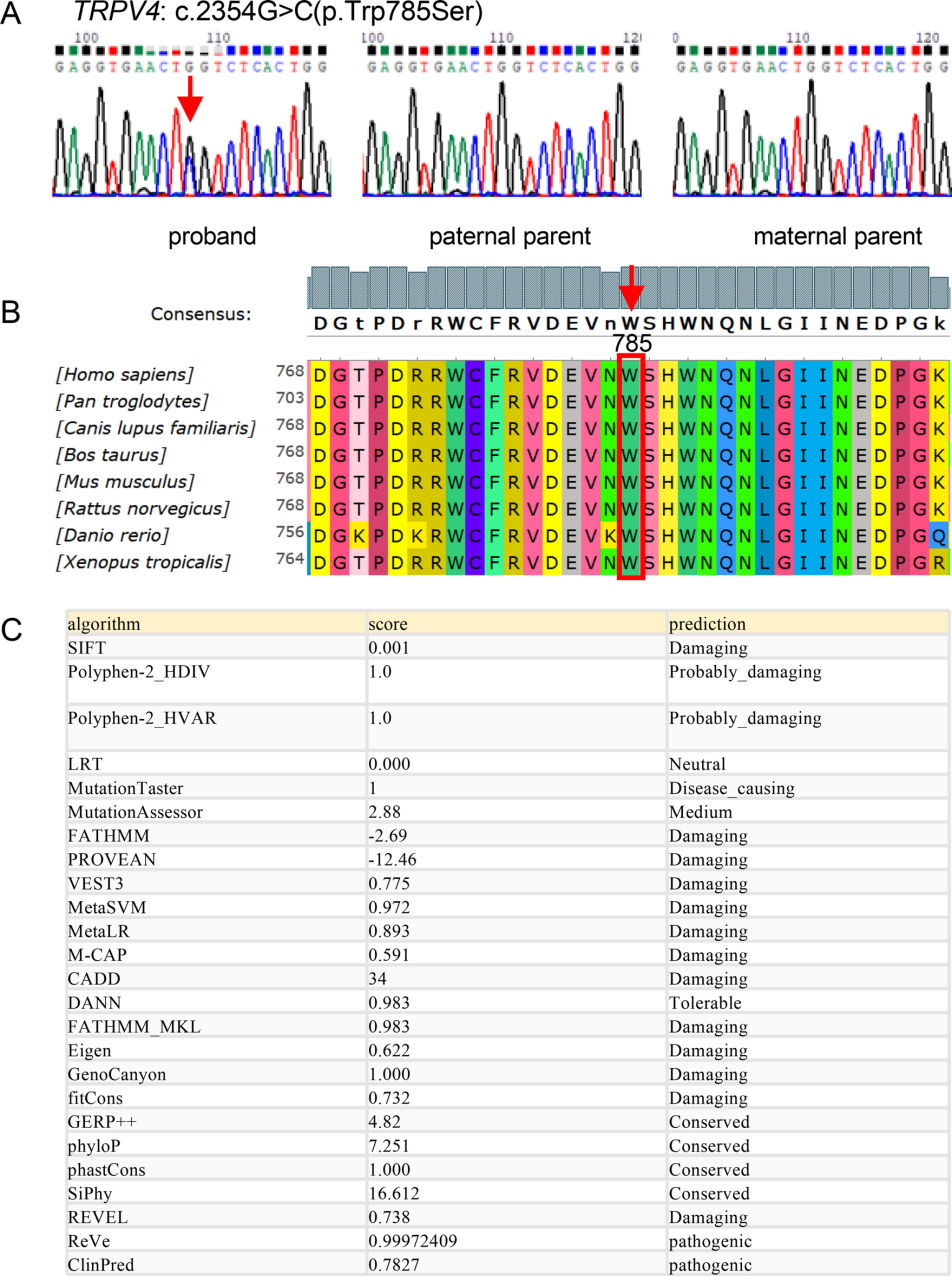
Mutation validation and pathogenicity analysis of *TRPV4*: c.2354G > C (p.Trp785Ser). (**A**) Validation of mutations in the proband and proband’s parents through Sanger sequencing; (**B**) Conservation analysis of the mutation site in different species; (**C**) Pathogenicity prediction of the mutation site

Whole-exome sequencing (WES) identified a heterozygous missense variant in the TRPV4 gene of the proband: c.2354G > C (p.Trp785Ser). This variant was subsequently validated by PCR and Sanger sequencing in the proband and both parents (Fig. 2A). The mutation was confirmed in the proband but absent in both parents, indicating a likely de novo origin. Nevertheless, the possibility of low-level parental germline mosaicism cannot be entirely excluded.

Conservation analysis across species demonstrated high conservation at this locus (Fig. 2B), further suggesting that mutations at this site could have a significant impact on the function of the TRPV4 protein. Subsequently, in silico missense prediction was performed to assess the pathogenicity of this variant, as depicted in Fig. 2C. Multiple prediction software tools indicated that this site is conserved and predicted to be damaging or pathogenic.

### Impact of *TRPV4*-W785S on protein structure and function

**Fig. 3:**
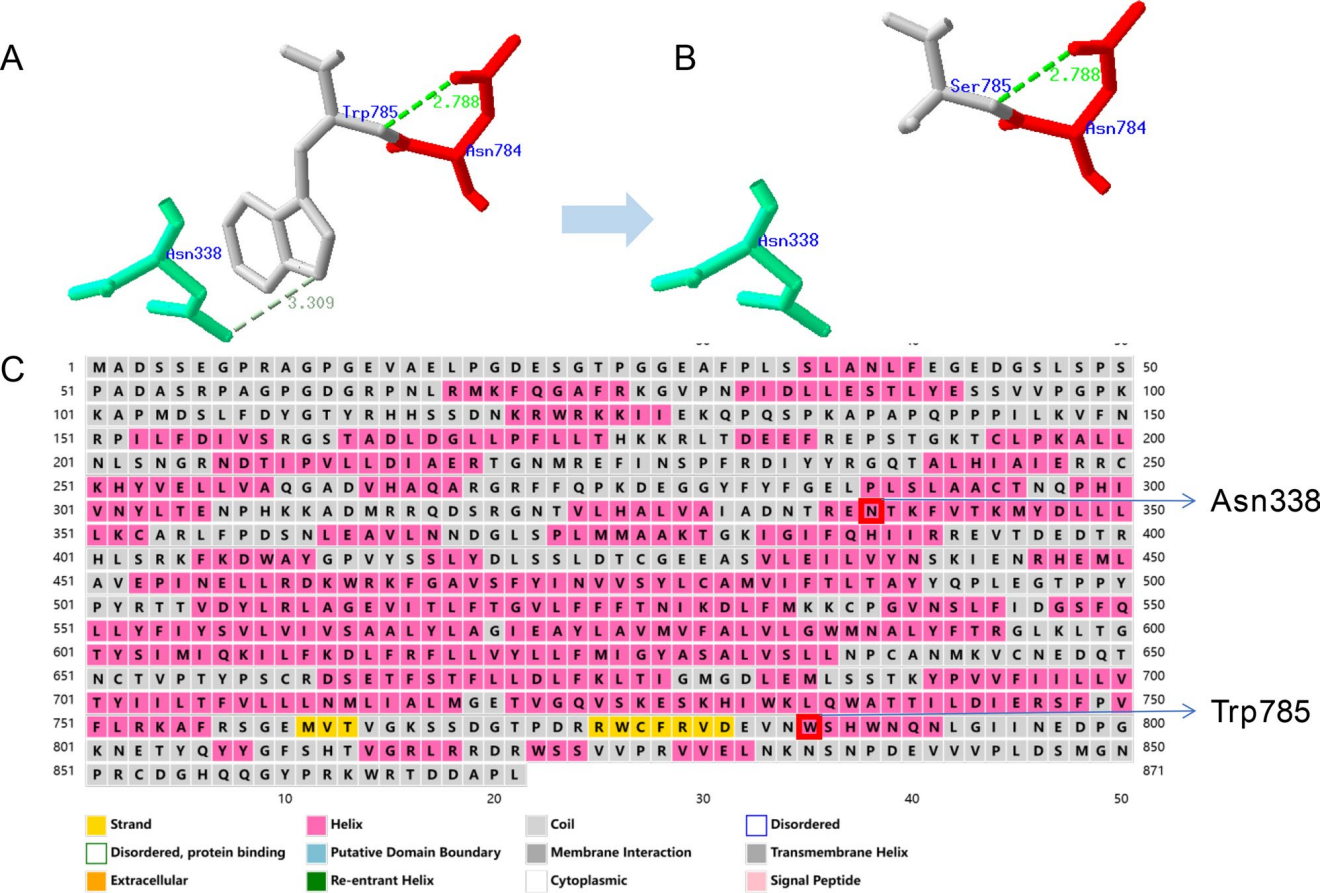
Structure prediction and functional analysis of *TRPV4*-W785S. (**A**) Protein tertiary structure prediction of TRPV4-Trp785; (**B**) Protein tertiary structure prediction of TRPV4-Ser785. (**C**) Secondary structure prediction of the TRPV4 protein

The *TRPV4*: c.2354G > C mutation results in the substitution of a hydrophilic tryptophan residue with a hydrophobic serine residue at position 785. The side chain of tryptophan contains a pyrrole ring and an aromatic ring structure, with the pyrrole ring portion possessing a polar amino group and a carboxyl group, which imparts hydrophilic properties to it. On the other hand, the side chain of serine is a relatively simple hydrophobic side chain, primarily composed of a methyl group and a hydroxyl group. This hydrophobic side chain tends to avoid contact with water, and therefore serine is often found in the hydrophobic core region of proteins, contributing to their structural stability [11]. As an ion channel protein, changes in the hydrophilicity of individual amino acids can potentially impact the protein’s ion permeability. Additionally, protein tertiary structure prediction of TRPV4 using SWISS-MODEL revealed a disruption of hydrogen bonding between tryptophan at position 785 and asparagine at position 338 due to the *TRPV4*: c.2354G > C mutation [12, 13]. Furthermore, secondary structure prediction of the TRPV4 protein (http://bioinf.cs.ucl.ac.uk/psipred/) indicated that both tryptophan at position 785 and asparagine at position 338 are located within an α-helix. The loss of their interaction may potentially affect the stability of the protein’s helical structure [14] (Fig. 3).

### Effect of *TRPV4*-W785S mutation on intracellular calcium and electrophysiology

**Fig. 4:**
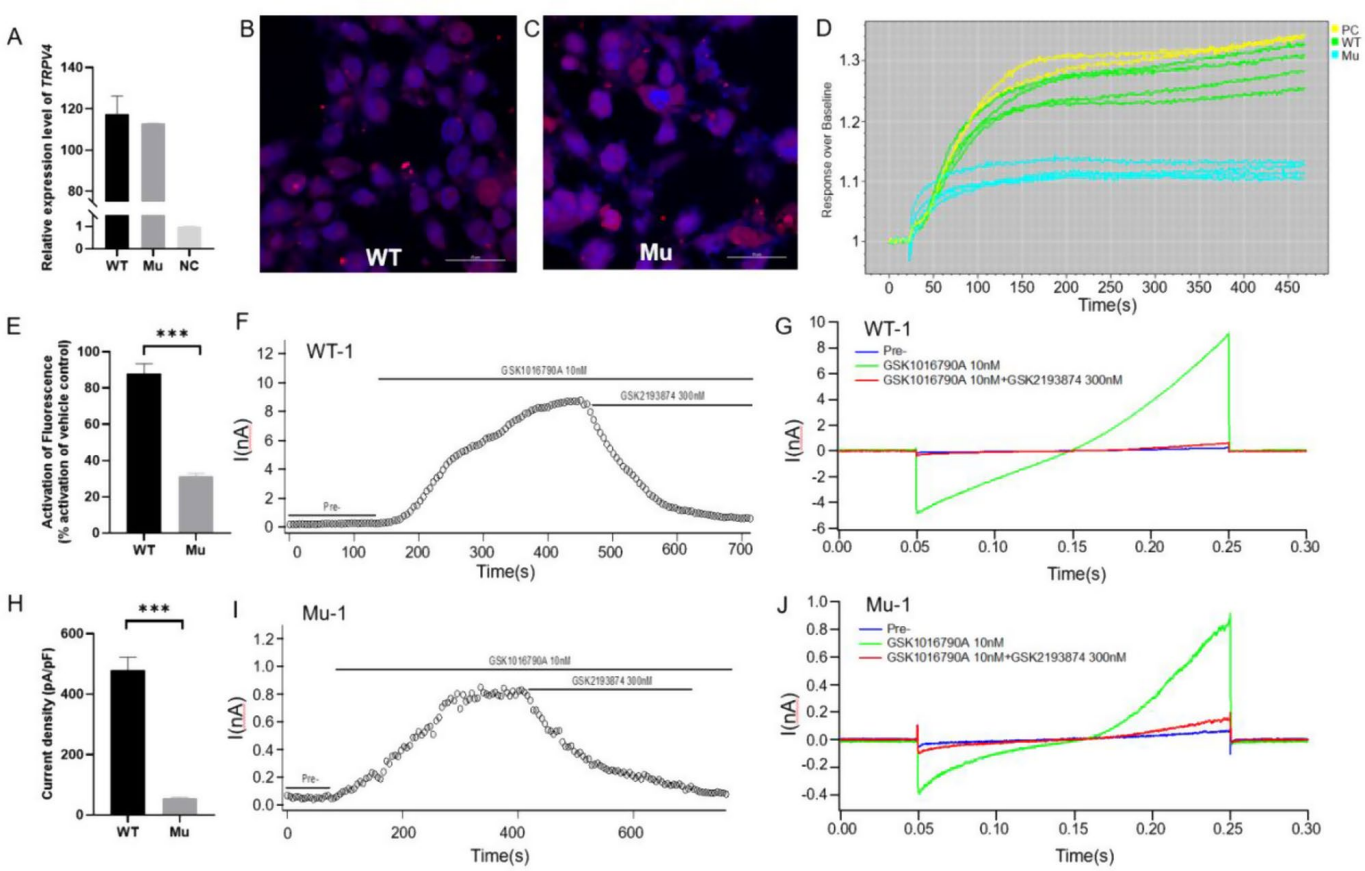
Functional assays on the effects of *TRPV4*-W785S on ion channels and calcium activity. **A**. Real time quantitative PCR results of HEK293 cells after transfected with TRPV4 vectors. *N* = 3, WT, wild type, Mu, mutant type, NC, negative control. **B**-**C**. Confocal microscopy images of HEK293 cells after 48 h after transfected with TRPV4 vectors. The red signal represents m-Cherry, blue represents DAPI. **D**. Response curve of stimulation with 10 nM agonist GSK1016790A in FLIPR assay, PC, positive control. **E**. Fluorescence activation efficiency of HEK293 cells transfected with two vectors compared to positive control, *n* = 4. **F**-**G**. Recording of TRPV4 ion channel current in HEK293 cells transfected with *TRPV4*-WT vector before and after stimulation with agonists and antagonists. **H**. The statistical results of current density showed activity of TRPV4 receptor channel in HEK293 cells transfected with *TRPV4*-WT and *TRPV4*-W785S vectors. **I**-**J**. Recording of TRPV4 ion channel current in HEK293 cells transfected with *TRPV4*-W785S vector before and after stimulation with agonists and antagonists. One-way ANOVA, ***P < 0.001, data were presented as mean ± SEM

We separately constructed *TRPV4*-WT and *TRPV4*-W785S vectors (Fig. S1) and transfected them into HEK293 cells. Real time quantitative PCR results showed that both vectors effectively transduced into HEK293 cells compared to the NC group (Fig. 4A). Confocal microscopy images also demonstrated successful expression of TRPV4-WT and TRPV4-W785S proteins (Fig. 4B-C), with blue representing DAPI-labeled cell nuclei and red indicating TRPV4 protein. The FLIPR results of detection of intracellular calcium showed that after stimulation with the agonist GSK1016790A, the HEK_*TRPV4*−WT_ group showed an average of 88% activation compared to the positive control (PC) group, while the HEK_*TRPV4*−W785S_ group only had an average of 31% activation (Fig. 4D-E) [15]. Detection of action potential (AP) of TRPV4 ion channel current by patch clamp on HEK293 cells transfected with vectors, results showed that 10 nM agonist GSK1016790A could activate the action potential of both HEK_*TRPV4*−WT_ and HEK_*TRPV4*−W785S_ cells, and 300 nM antagonist GSK2193874 could convert AP to resting level (Fig. 4F-J) [16, 17]. The maximum membrane current of the HEK_*TRPV4*−WT_ reached about 9nA (Fig. 4F-G), while the maximum membrane current of the HEK_*TRPV4*−W785S_ was only about 0.8nA (Fig. 4I-J). The current density of HEK_*TRPV4*−W785S_ was significantly reduced compared to HEK_*TRPV4*−WT_, indicating weaker electrical activity and response to agonists (Fig. 4H).

## Discussion

### Clinical and radiological spectrum of *TRPV4*-related dysplasias

TRPV4-related skeletal dysplasias encompass a clinical spectrum, including metatropic dysplasia (MD), spondylometaphyseal dysplasia, Kozlowski type (SMDK), and autosomal dominant brachyolmia (BO) [5], which share overlapping radiographic features but differ in severity, distribution of skeletal involvement, and extra-skeletal complications [18]. The proband presents with postnatal-onset skeletal abnormalities, including spinal changes, mild scoliosis, pectus carinatum, metaphyseal irregularities, and joint pain in multiple large joints. These features are inconsistent with MD, which typically manifests as a severe, progressive disorder beginning in infancy, with hallmark findings such as a narrow thoracic cage, “dumbbell-shaped” long bones, and wafer-like platyspondyly. Moreover, MD often includes a prominent facies, respiratory compromise, and coccygeal tail, none of which are observed in this case. Similarly, BO is characterized by isolated vertebral flattening (platyspondyly) with minimal metaphyseal or epiphyseal changes. While the proband does exhibit platyspondyly and overfaced pedicles—radiographic findings occasionally seen in both BO and SMDK—the presence of metaphyseal flaring, joint space narrowing, and knee deformities suggests a diagnosis beyond BO, which lacks appendicular bone involvement.

In contrast, the proband’s features more closely align with SMDK, which typically presents in early childhood with short trunk stature, platyspondyly, overfaced pedicles, and metaphyseal irregularities of the long bones. While short stature is not observed in this case, phenotypic variability has been reported in autosomal dominant TRPV4 disorders [8]. The presence of pectus carinatum without respiratory limitation, spinal narrowing with possible neural symptoms, and joint deformities primarily affecting knees and elbows supports compatibility with the clinical spectrum of SMDK. Radiographically, the combination of vertebral body narrowing, overfaced pedicles, flaring of the ilia, and degenerative changes in long bone joints further supports this interpretation.

Therefore, while the patient’s phenotype does not exhibit the full classic picture of any single entity, the constellation of findings—especially in contrast to the more severe or restricted patterns of MD and BO—suggests a close alignment with the milder end of the SMDK spectrum, consistent with a TRPV4-related skeletal dysplasia.

### Genetic and structural characterization supports pathogenicity

Although the p.W785S variant is listed in the Human Gene Mutation Database (HGMD) as disease-causing mutation (DM), we noted that the cited reference linked to this variant in the database does not actually mention this substitution, which raised some confusion [19]. Further searches in other public databases also failed to retrieve any published literature reporting this variant. To our knowledge, the present study provides the first definitive documentation of this variant, including structural modeling, functional validation, and clinical correlation.

According to ACMG guidelines [20], population screening confirmed the absence of the variant in gnomAD (PM2), and pedigree analysis supports the cosegregation of the variant with disease in affected family members. Sanger sequencing revealed that neither parent carries the variant, suggesting a presumed de novo origin (PM6). Cross-species sequence alignment showed complete conservation of the Trp785 residue across vertebrates, highlighting its functional significance. This residue is located within the C-terminal cytoplasmic tail of *TRPV4*, a region associated with protein-protein interactions and channel gating regulation. Critically, a missense change at the same codon, c.2355G > T (p.Trp785Cys), has been reported as pathogenic with co-segregation in a multigenerational family exhibiting *TRPV4*-related phenotypes; however, no functional assays were performed in that study [21]. This finding supports PM5 and reinforces the clinical relevance of this residue. In addition, a single-nucleotide deletion at the same locus resulting in a frameshift (p.Trp785CysfsTer48) is cataloged in gnomAD; as of 30 June 2025, it was observed once among 44,878 East Asian alleles (allele frequency 0.00002228), with no germline clinical classification provided.

Structural modeling revealed that substitution of the hydrophilic Trp with the smaller, more hydrophobic Ser may disrupt the hydrogen bonding network within the channel, specifically abolishing a key interaction with Asn338. Given that both residues reside in α-helices, this interaction likely contributes to the structural stability of the TRPV4 channel. Disruption of this hydrogen bond may induce local conformational changes, potentially impairing ion channel gating or membrane trafficking.

### Functional impairment of TRPV4 W785S suggests an atypical pathogenic mechanism

Functional analyses in HEK293 cells revealed that wild-type and W785S TRPV4 were expressed at comparable protein levels. However, GSK1016790A-evoked intracellular Ca²⁺ influx was reduced by approximately 65%, and patch-clamp recordings demonstrated > 90% reduction in whole-cell current in W785S-expressing cells compared to wild-type, fulfilling the ACMG criterion for strong functional evidence of a damaging effect (PS3). This loss-of-function profile contrasts with the classical gain-of-function mechanism reported in most TRPV4-related skeletal dysplasias, suggesting an atypical pathogenic pathway.

This hypofunctional phenotype deviates from the commonly reported gain-of-function mechanism and may explain the relatively milder skeletal manifestations in our patient [5, 22]. It supports emerging evidence that *TRPV4* mutations can produce phenotypic heterogeneity through distinct functional consequences, including partial loss of function [23]. Such impairment may interfere with downstream calcium-dependent signaling pathways, ultimately affecting chondrogenesis and bone development [24].

This case highlights the need to consider alternative mechanisms beyond channel hyperactivity in *TRPV4*-associated disorders and underlines the importance of functional studies in clarifying mutation-specific effects. Future work using patient-derived chondrocytes or iPSC-based models will help further elucidate the skeletal impact of *TRPV4* hypofunction [25].

## Conclusion

In this study, we identified a *TRPV4* missense variant p.W785S, in a patient with clinical and radiological features consistent with SMDK. Although this variant has been included in the HGMD and annotated as DM, the cited reference does not describe p.W785S, and no additional reports were retrieved from other public variant databases. To our knowledge, this represents the first report to provide functional validation and clinical correlation for p.W785S. The variant fulfills multiple ACMG/AMP criteria—PS3 (strong), PM2 (moderate), PM5 (moderate), and PM6 (moderate)—supporting its classification as a novel pathogenic *TRPV4* mutation. Functionally, this variant leads to reduced TRPV4 channel activity, suggesting a hypofunctional mechanism. Functional assays demonstrated a marked reduction in TRPV4 channel activity, indicating a hypofunctional mechanism that contrasts with the canonical gain-of-function effects observed in most *TRPV4*-related skeletal dysplasias. This functional impairment may account for the relatively mild skeletal phenotype in the patient and contributes to the broader understanding of phenotypic heterogeneity in *TRPV4*-associated disorders.

## Electronic supplementary material

Below is the link to the electronic supplementary material.

Supplementary Material 1

## Data Availability

The datasets generated and/or analyzed during the current study are available from the corresponding author upon reasonable request.
