# Supplementary material for: A novel TRPV4 variant in spondylometaphyseal dysplasia, kozlowski type reveals a previously unreported loss-of-function mechanism
Source: Orphanet J Rare Dis. 2025 Nov 12;20:575. doi: 10.1186/s13023-025-04070-y (PMC12613735; doi:10.1186/s13023-025-04070-y)
Supplement: Supplementary file 1 — Supplementary Material 1 [file 13023_2025_4070_MOESM1_ESM.docx]

Supplementary Figure 1

**FIGURES1|** Plasmid maps of TRPV4-WT(A) and TRPV4-W785S(B).
